# Typology of Battery Cells – From Liquid to Solid Electrolytes

**DOI:** 10.1002/advs.202303985

**Published:** 2023-09-26

**Authors:** Sudeshna Sen, Felix H. Richter

**Affiliations:** ^1^ Institute of Physical Chemistry Justus‐Liebig‐University Giessen Heinrich‐Buff‐Ring 17 35392 Giessen Germany; ^2^ Center for Materials Research (ZfM) Justus‐Liebig‐University Giessen Heinrich‐Buff‐Ring 16 35392 Giessen Germany; ^3^ Present address: WMG University of Warwick Coventry CV4 7AL UK

**Keywords:** battery, hybrid electrolyte, liquid electrolyte, polymer electrolyte, solid electrolyte

## Abstract

The field of battery research is bustling with activity and the plethora of names for batteries that present new cell concepts is indicative of this. Most names have grown historically, each indicative of the research focus in their own time, e.g. lithium‐ion batteries, lithium‐air batteries, solid‐state batteries. Nevertheless, all batteries are essentially made of two electrode layers and an electrolyte layer. This lends itself to a systematic and comprehensive approach by which to identify the cell type and chemistry at a glance. The recent increase in hybridized cell concepts potentially opens a world of new battery types. To retain an overview of this dynamic research field, each battery type is briefly discussed and a systematic typology of battery cells is proposed in the form of the short and universal cell naming system ^AAM^XEB^CAM^ (AAM: anode active material; X: L (liquid), G (gel), PP (plasticized polymer), DP (dry polymer), S (solid), H (hybrid); EB: electrolyte battery; CAM: cathode active material). This classification is based on the principal ion conduction mechanism of the electrolyte during cell operation. Even though the presented typology initiates from the research fields of lithium‐ion, solid‐state and hybrid battery concepts, it is applicable to any battery cell chemistry.

## Graphical abstract

Since battery research has matured to the exploration of increasingly complex electrolyte compositions, it appears timely to propose a unified typology of battery cells and accompanying short notation. Its adoption is expected to aid with clarity, focus, and building bridges between different strands of battery research.

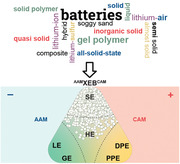

## 1 Introduction

Naming immediately comes to mind when inventing a new technology, as each invention also calls for a new name. When screening the academic literature in the battery field, the plethora of names used is indicative of an exciting, dynamic, and thriving research field. However, this makes it unduly complicated to identify at a glance the type of cell system investigated in the respective publication. The simple reason is that battery researchers so far miss a universal and systematic methodology that classifies batteries according to their working principle in terms of ion conduction mechanism and cell chemistry.

In their recent article, Frith, Lacey and Ulissi request researchers to sharpen the focus and help bridge the gap between academic and industrial research.^[^ ^1^ ^]^ As different cell types have reached different levels of maturity, the highest achieved battery performance is strongly dependent on the cell type. For instance, lithium‐ion batteries with liquid electrolytes (LEs) have reached a much higher technology readiness level than for example solid‐state or hybrid battery concepts.^[^ ^2^ ^]^ Therefore, it is important to know the cell type in order to evaluate the progress over the state‐of‐the‐art. Considering the above, it appears timely to propose a simple and uniform classification system encompassing all battery types.

Conceptually, every battery is simply made of three layers: positive electrode layer, electrolyte layer, negative electrode layer. The electrolyte layer is solely ion conducting, serves to separate the electrodes electronically and is sandwiched between positive and negative electrode layers. Over time, researchers developed different kinds of battery chemistries comprising ion intercalation reactions,^[^ ^3^ ^]^ conversion reactions^[^ ^4^ ^]^ and different types of electrolytes. In order to describe battery types in daily communication, convenient terms, such as lithium‐ion batteries, lithium‐sulfur batteries, lithium‐air batteries, etc., were established, highlighting the initial research focus on electrode development. However, these descriptors do not contain information about the electrolyte and use of a conventional LE is typically implied.

As research attention has recently expanded to diversifying the electrolyte chemistry, (all‐)solid‐state batteries and hybrid cells have become more prominent.^[^ ^5^, ^6^ ^]^ However, there are several different types of electrolytes used in (all‐) solid‐state batteries and some electrolytes rely on the help of a liquid additive during cell operation. In line with this, the liberal use of descriptive terms for cases combining different types of electrolytes and materials, such as “hybrid”,^[^ ^7^ ^]^ “composite”,^[^ ^8^ ^]^ “quasi solid”,^[^ ^9^ ^]^ “semi solid”,^[^ ^10^ ^]^ or “almost solid”,^[^ ^11^ ^]^ leave room for ambiguity about the electrolyte composition and ion conduction mechanism. In addition, these terms specify neither the electrode composition nor the full cell chemistry. Ideally, a unified and systematic battery terminology identifies each battery composition clearly, uniquely and simply as a certain cell type. Therefore, we wish to address the question: what defines the battery type?

This article gives an overview of different types of battery cells, evaluates their performance to date and proposes a general classification method that distinguishes different cell types systematically. The basis for classification is the main ion conduction mechanism of the electrolyte. In addition, the proposed short notation for full cells also states the chemistry of the electrodes. It is applicable, in principle, to many types of electrochemical cells, but is predominantly exemplified in this article by cells using lithium ions as mobile charge carrier.

## 2 Proposed Classification of Batteries

### 2.1 A universal Battery Classification based on the Ion Conduction Mechanism

The chemistry and physical properties of the electrolyte significantly influence battery manufacture and performance. An ideal electrolyte should exhibit unity transference number and high ionic conductivity at least comparable to that of liquid electrolytes. Both parameters are important for faster ion transport and the decrease of internal cell resistance. In addition, interface‐related challenges, such as dendrite formation, instability of electrolytes with electrodes, and sluggish electrode kinetics play an important role. In particular, the ion conduction mechanism, ionic conductivity, transference number, mechanical properties, thermal properties, surface properties, and electrochemical windows are crucial parameters governing the advantages and disadvantages of each electrolyte type, which also strongly influence battery performance and manufacture.^[^ ^12^, ^13^ ^]^ Presently, electrolytes are classified simply according to their composition and physical state of matter: for example, the terms solid, inorganic solid, ceramic, solid polymer, soggy sand, semi‐solid, quasi‐solid, almost solid, gel polymer, composite, hybrid, and liquid are used to describe and categorize electrolytes. However, these descriptors leave room for interpretation as to the predominant ion conduction mechanism that occurs in the electrolyte.

**Figure 1:**
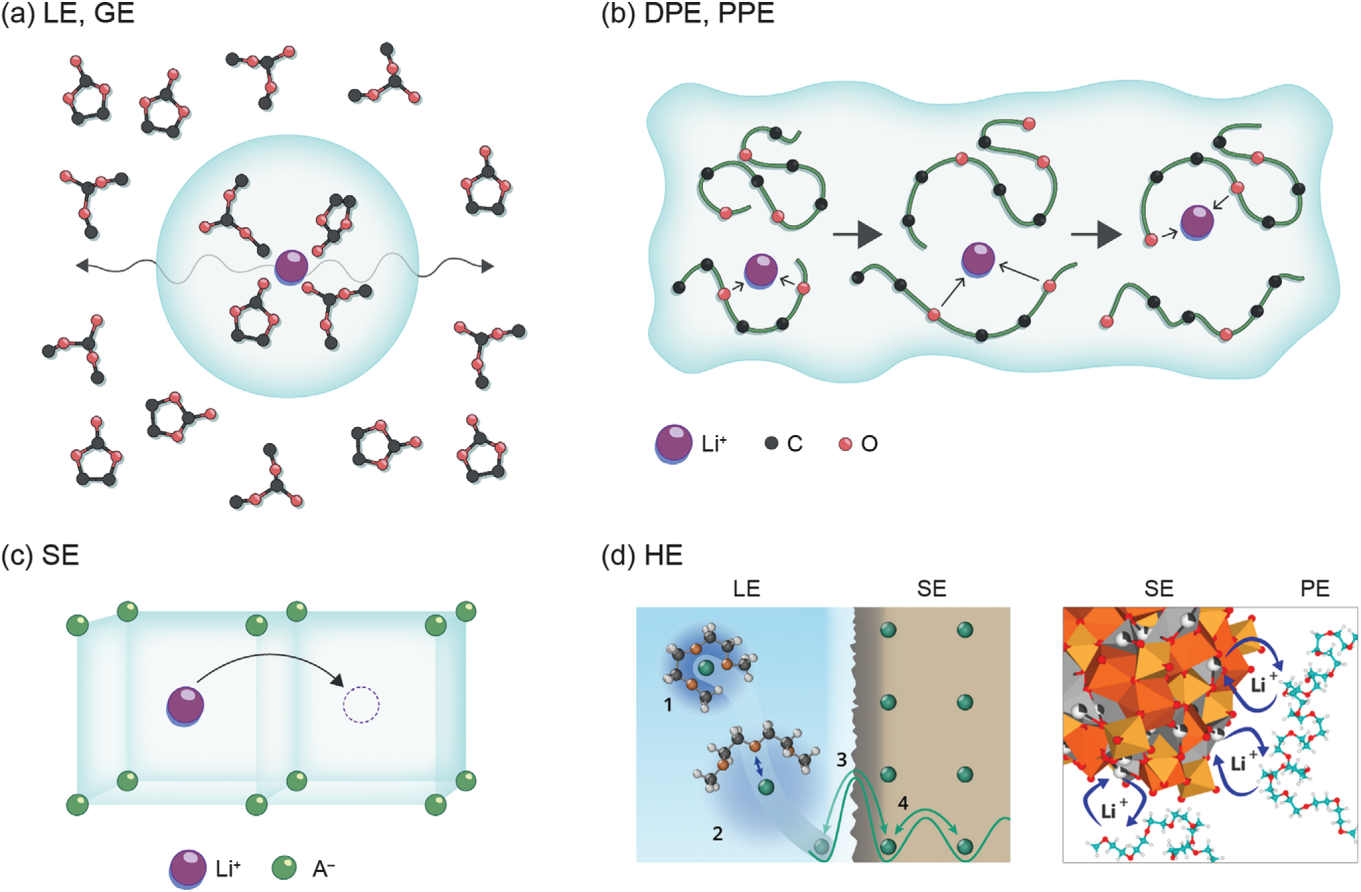
Schematic of different types of electrolytes and ion conduction mechanisms: a) mobile ion‐solvent complexes in LEs and GEs^[^ ^14^, ^15^, ^16^, ^17^ ^]^ b) polymer chain segmental motion in DPEs and PPEs^[^ ^18^ ^]^ c) ion transport by vacancies and interstitial sites in SEs^[^ ^18^, ^19^, ^20^ ^]^ and d) interfaces in HEs, for which schematics of a LE‐SE‐interface (left, adapted under the terms of the CC‐BY license,^[^ ^21^ ^]^ Copyright 2020, Springer Nature) and a PE‐SE‐interface (right, adapted with permission,^[^ ^22^ ^]^ Copyright 2019, American Chemical Society) are shown.

In our view, the most relevant parameter to classify electrolytes is their ion conduction mechanism. Following this perception, we suggest the following classification of electrolytes into four types of predominant ion conduction mechanisms: 1) mobile ion‐solvent complexes define a liquid electrolyte (LE) or gel electrolyte (GE), 2) ion transport through polymer chain segmental motion defines a dry polymer electrolyte (DPE) or plasticized polymer electrolyte (PPE), 3) ion transport by vacancies and interstitial sites defines a solid electrolyte (SE), and 4) a hybrid electrolyte (HE) is defined as any combination of the above in which at least two types of conduction mechanism are present in the electrolyte or cell. **Figure** **1**a–c depicts these electrolyte types and their respective ion transport mechanisms. A characteristic feature of the HE is that it includes ion transfer from one electrolyte type to another, as depicted in two examples in Figure 1d. Most electrolytes easily categorize within the proposed classification system with only few borderline cases whose properties strongly depend on the respective composition and operation temperature.

**Figure 2:**
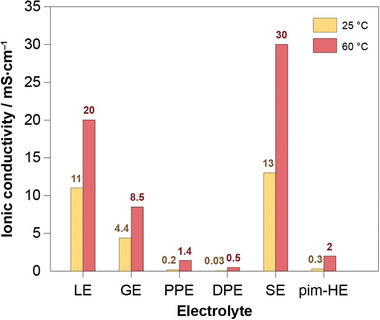
Ionic conductivity values of examples for each class of electrolyte – LE: 1 m LiPF_6_ in ethylene carbonate‐dimethyl carbonate,^[^ ^17^] GE: mixture of 10 wt.% cross‐linked poly(methyl methacrylate) and 90 wt.% LE,^[^ ^23^ ^]^ PPE: polyethylene oxide (PEO):LiTFSI:Pyr_14_TFSI in ratio 20:2:2,^[^ ^24^ ^]^ DPE: PEO‐LiClO_4_,^[^ ^25^ ^]^ SE: Li_10_GeP_2_S_12_,^[^ ^26^ ^]^ and particle‐in‐matrix (pim) HE: mixture of Li_1.3_Al_0.3_Ti_1.7_(PO_4_)_3_ fibers, poly[bis(2‐(2‐methoxyethoxy)ethoxy)‐phosphazene] and lithium(4‐styrenesulfonyl)‐(trifluoromethanesulfonyl)imide.^[^ ^27^ ^]^ Note: these examples demonstrate high conductivities achieved within each electrolyte type and the conductivity of other examples may deviate significantly from the values shown here.

Typical ionic conductivity values for each electrolyte class with specific examples are summarized in **Figure** **2**. It is evident that the large spread of achieved conductivities over two orders of magnitude results in vastly different battery performance. As the conduction mechanism and type of the electrolyte are fundamental to how the battery functions, we extend the same principles to the classification of battery cells. In our proposed terminology, the corresponding types of batteries employing each respective class of electrolyte are noted accordingly: 1) liquid electrolyte battery (LEB) or gel electrolyte battery (GEB), 2) dry polymer electrolyte battery (DPEB) or plasticized polymer electrolyte battery (PPEB), 3) solid electrolyte battery (SEB), and 4) hybrid electrolyte battery (HEB). For example, a LEB describes a cell with liquid electrolyte, a prominent example of which is commonly known as the lithium‐ion battery.

**Figure 3:**
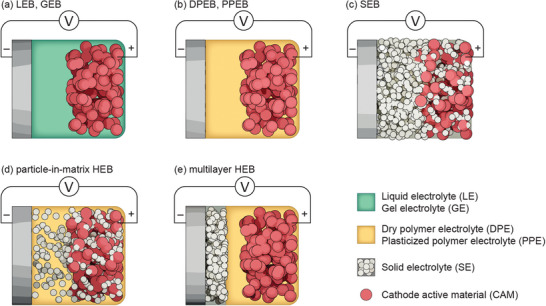
Schematics of batteries made of a metal negative electrode (for example lithium), a positive electrode containing cathode active material (CAM) particles and an electrolyte, forming either a a) LEB or GEB, b) DPEB or PPEB, c) SEB, d) particle‐in‐matrix HEB, or e) multilayer HEB. Note: even though the SE‐PE combination depicted in the HEB images is a commonly investigated combination, it serves as an example here and in principle any combination of electrolytes may be prepared in the form of a particle‐in‐matrix or multilayer HEB.

**Figure** **3** summarizes the different battery types distinguished in our proposed cell classification. The schematics exemplarily contain one metal electrode and one composite electrode each. For LEBs, GEBs, PEBs and SEBs, there is only one electrolyte type throughout the entire battery. In order to reduce complexity and keep the classification as simple as possible, a HEB simply encompasses any cell, which uses a combination of different electrolyte types, independent of how they are assembled in the cell. We have previously presented an overview of possible hybrid electrolyte architectures,^[^ ^15^ ^]^ two generalized examples of which are shown exemplarily in Figure 3d in the form of particle‐in‐matrix and in Figure 3e in the form of multilayer assemblies. Typically, the combination of two electrolyte types in one cell creates additional interfaces at which the mobile ions need to transfer from one electrolyte to the other.[^21^] The following sections briefly describe the respective state‐of‐the‐art for each type of electrolyte and battery.

### 2.2 Liquid Electrolyte Battery and Gel Electrolyte Battery

#### 2.2.1 Liquid Electrolyte Battery (LEB)

LEs are made by dissolving salt in liquid solvents with low molecular weight and viscosity. For example, lithium‐ion batteries typically use 1 m solutions of lithium salts (LiPF_6_, LiTFSI, etc.) dissolved in non‐aqueous organic solvents, such as ethylene carbonate, dimethyl carbonate, etc. Lithium salt solutions in other aprotic organic solvents or ionic liquids^[^ ^28^, ^29^ ^]^ have also been explored widely as LEs. In exceptional cases, aqueous electrolytes are also used.^[^ ^30^ ^]^ In LEs, both solvated cations as well as solvated anions are mobile species. Typically, the ionic conductivity of LEs reaches 10^−2^ S cm^−1^.^[^ ^15^ ^]^

In LEs, ion transport requires the migration of solvated ions that are created by dissociation and solvation of a conducting salt in a solvent.^[^ ^31^ ^]^ During the process of solvation, solvent molecules coordinate to the ions and the ion migrates along with a solvation sheath. The fast exchange of solvated ions and solvating compounds creates a uniform ionic environment.^[^ ^16^ ^]^ Therefore, ion transport is diffusion‐limited and coupled with the viscosity of the liquids.^[^ ^3^, ^4^ ^]^ Ion conduction is facilitated by reduced viscosity of the solvent (increase in mobility of ions) and dissociation of ion pairs (increase in the concentration of mobile ions).

Even though LEBs present the most advanced technology to date, lithium LEBs with organic electrolytes may suffer from thermal runaway, leakage of LE out of the cell, drying up of LE in the cell, side reactions between lithium metal electrodes and organic electrolytes, forming an unstable interface leading to dead lithium formation and irreversible capacity loss.^[^ ^32^ ^]^ Cell aging tests carried out with Tesla's 18650 lithium‐ion cells confirmed that the capacity fade and aging of the cell (rise in impedance) is strongly related to the composition of solid electrolyte interface (SEI) formed with a graphite anode.^[^ ^33^ ^]^ Following such reports, current LEB research focuses on optimizing electrolytes that can produce uniform SEI, uniform ion flux, and favorable SEI composition. More advanced electrolyte engineering is proposed, targeting SEI stabilization. Electrolyte formulation includes optimization of additives, solvent ratio modification, and high salt concentrated electrolytes by using fluorinated solvent and salt additives to optimize SEI formation on the graphite or lithium anode during cycling.^[^ ^34^ ^]^ The oxidation of electrolyte with high voltage cathode is another crucial factor for determining capacity fading.

#### 2.2.2 Gel Electrolyte Battery (GEB)

The combination of liquid and polymer provides several intermediate cases dependent on composition and miscibility. With increasing polymer content, the properties of the mixture change continuously from liquid polymer solution, via gel polymer (polymer in solvent), and plasticized polymer (solvent in polymer), to dry polymer.^[^ ^18^ ^]^ As the transition between all of these cases is continuous, careful classification must be made on a case‐by‐case basis for polymer‐containing electrolytes. In general, gels are formed by entrapping liquid in a miscible polymer matrix, often a cross‐linked polymer network.^[^ ^35^ ^]^ Thus, a GE combines the properties of liquids and polymers: cohesive structure of polymers and conduction properties of liquids.^[^ ^36^, ^37^ ^]^ The ion transport occurs predominantly through the entrapped LE provided that the gel contains a high content of liquid solvent.^[^ ^38^ ^]^

In general, GEs have comparable ionic conductivity and conduction properties to those of the corresponding LEs, but due to their distinctive mechanical properties, they are clearly distinguishable from LEs, which is why we suggest distinguishing the type of GEBs. In general, polymers explored to form gels are polyacrylonitrile (PAN), polyvinylidene fluoride (PVDF), or poly(methyl methacrylate) (PMMA) swollen with molecular solvents or plasticizers such as propylene carbonate, ethylene carbonate, dimethylformamide, or tetraglyme.[^37^] The methyl methacrylate‐acrylonitrile‐styrene ter‐polymer takes up ethylene carbonate, diethyl carbonate and LiClO_4_ to form a GE for cells using lithium and LiNi_0.83_Co_0.17_O_2_.^[^ ^39^ ^]^

For example, Lee et al.^[^ ^40^ ^]^ presented a plastic‐crystal‐embedded elastomer electrolyte comprising succinonitrile (SN)‐butyl acrylate (BA)‐poly(ethylene glycol) diacrylate (PEGDA) lithium bis(trifluoromethanesulfonyl)imide (LiTFSI). The elastic gel showed ionic conductivity of 1.1 mS cm^−1^ at 20 °C. This GE is evaluated in a full cell with NMC cathode exceeding specific energy of 410 Wh kg^−1^ (Li | SN‐LiTFSI‐PEGDA‐BA‐LiTFSI | NMC). Another example of a GE comprises pyrolidinium‐based ionic liquid‐LiTFSI entrapped inside a cross‐linked polymer network of poly(acrylonitrile) or poly(AN‐co‐MSMA) network (AN: acrylonitrile; MSMA: 3‐(trimethoxysilyl)propyl methacrylate), where network modification leads to variable conductivity, mechanical properties and ion diffusion coefficient or conduction mechanism. One common property for all of the above gels is that ion transport is guided by entrapped liquid and the polymer host contributes to ionic interaction and hence number density of mobile charge and ionic mobility.

Potential drawbacks of the GEB are similar to those of the LEB as there may still be a chance of electrolyte leakage. The in‐situ polymerization methodology may improve the mechanical properties and battery assembly. However, ionic conductivity of gels often decreases when using the in‐situ polymerization of polymer in liquid approach. The key advantage of the system is the ease of processing and manufacturing potential.

### 2.3 Dry Polymer Electrolyte Battery and Plasticized Polymer Electrolyte Battery

#### 2.3.1 Polymer Electrolyte Battery (DPEB)

In contrast to LEs and GEs, PEs use complexes of polymers and conducting salt for ion conduction. Although they are sometimes called “solid polymer electrolyte”, here, we denote this class simply as PE, as the physical state depends on the temperature and may be very soft or even liquid during cell operation. In 1973, the concept of PEs was first proposed by Fenton et al. who observed ionic conductivity improvement in polyethylene oxide (PEO) when dissolving metal salt complexes. A common example of DPEs is polyethylene oxide lithium bis(trifluoromethanesulfonyl)imide (PEO‐LiTFSI). Alternatively, other polymers with polar functional, a blend of polymers,^[^ ^41^ ^]^ copolymers, block copolymers or polyelectrolytes^[^ ^42^ ^]^ are used as polymer host for lithium salts. The achievable ionic conductivity in polymer salt complexes is in the range of 10^−6^ to 10^−4^ S cm^−1^ at room temperature.

The PE primarily distinguishes from the LE in ion conduction mechanism. In the amorphous state of polymers, lithium ions coordinate to polymer chains by electron‐donating groups of polymer chains (for example, ether oxygens in PEO). Such complex coordination leads to the separation of ion pairs of salts, enhancing salt dissociation. Above the glass transition temperature of the polymer, the polymer undergoes local segmental motion and free volume is created. Ion movement from one coordination site to another is assisted by segmental motion of the polymer.^[^ ^43^ ^]^ For perfect complexation, the lattice energy of the salt should be low, and the polymer matrix should have a high dielectric constant.^[^ ^44^ ^]^

An outstanding example of a DPEB was reported by Hovington et al., where polyether‐lithium bis (trifluoromethanesulfonyl) imide (LiTFSI) is used as a polymer separator with LiFePO_4_ cathode in the configuration (Li | PEO‐LiTFSI | LiFePO_4_).^[^ ^45^ ^]^ Another interesting example are so‐called single‐ion conductors.^[^ ^41^, ^42^, ^46^ ^]^ For example, Bouchet et al. reported poly(styrene trifluoromethane‐sulphonyl imide of lithium)‐block‐linear poly(ethylene oxide) based electrolyte to show ionic conductivity of 1.3×10^−5^ S cm^−1^ at 60 °C.^[^ ^42^ ^]^ In this case, the counterion of the conducting species is grafted to the block‐copolymer backbone.

PEs are commonly applied to create dimension stability of the electrolyte layer, free‐standing films, and to increase safety over LEs. They have been successfully applied with a lithium metal anode in a commercial product.^[^ ^45^ ^]^ Fine‐tuning of the polymer backbone and amorphous structure leads to a wide range of PEs with different properties. Currently, zwitterion structures and self‐healing polymers are explored as DPEBs. These prototypical examples of DPEs do not contain liquids or other additives as plasticizers.

#### 2.3.2 Plasticized Polymer Electrolyte Battery (PPEB)

Plasticizers are additives that increase polymer mobility, leading to decreased glass transition temperature and increased polymer chain dynamics.^[^ ^47^, ^48^ ^]^ Such plasticized polymers are similar to gels, but the plasticizer content is typically lower. Recently, Bai et al.^[^ ^49^ ^]^ reported high salt content plasticizer made of acetonitrile and LiTFSI for poly(dimethyl(methacryloyloxy)methyl phosphonate (PMAPC_1_) polymers to achieve ionic conductivity of 1.6 mS cm^−1^ at 100 °C. The plasticizer enhances segmental motion of polymer chains, for example, acetonitrile doping decreases the glass transition temperature of PMAPC_1_ and enhances salt dissociation and ion mobility, which speeds up ion transport through the polymer matrix. As long as the polymer matrix is the predominant mode of conduction and the added plasticizer merely acts to mobilize polymer chains, this type of cells forms part of PPEBs. For example, Bai et al. assembled a PPEB as Li | 1 PMAPC1 + 2 LiTFSI + 1 AN | LiFePO_4_, which exhibits a specific capacity of about 147 mAh g^−1^ in the first discharge cycle with capacity retention of 91.4% after 500 cycles, along with stable SEI characteristics.^[^ ^49^ ^]^

By use of the plasticizer, the ionic conductivity is enhanced over DPEs, while being less prone to leakage and thermal runaway than LEs or GEs. However, plasticization with solvent often softens the polymer network, sacrificing mechanical strength. The interface characteristics at anode and cathode are similar to those of DPEs. It is usually quite difficult to distinguish PPEs from GEs as the transition between the two cases is continuous and depends on the content of liquid/additive/plasticizer, requiring a careful classification of the system on a case‐by‐case basis.

### 2.4 Solid Electrolyte Battery (SEB)

SEs conduct ions through an ordered anion structure without requiring salt additives. Several inorganic SEs are reported for oxides, thiophosphates and other ionic compounds, some reaching conductivities beyond 10 mS cm^−1^ at room temperature with near to unity transference number. Common examples are LISICON (lithium superionic conductor),^[^ ^50^ ^]^ garnet‐type,^[^ ^51^ ^]^ NASICON (sodium superionic conductor),^[^ ^52^ ^]^ lithium nitride,^[^ ^53^ ^]^ lithium borohydrides,^[^ ^54^ ^]^ perovskites^[^ ^55^ ^]^ and mixed halides,^[^ ^19^ ^]^ argyrodite‐type electrolytes (Li_6.6_Si_0.6_Sb_0.5_S_5_I, Li_6.6_Ge_0.6_P_0.4_S_5_I, Li_5.5_PS_4.5_Cl_1.5_),^[^ ^26^, ^56^, ^57^, ^58^ ^]^ sulfide‐based solid solutions (Li_4_ *_x_* Sn_1−_ *_x_* S_2_)^[^ ^59^ ^]^ and their structure‐equivalent derivatives. Increasing ionic conductivity (up to 24 mS cm^−1^) can be achieved by structural and compositional tuning within a given family of structures.^[^ ^26^ ^]^

In general, ion transport in SEs is governed by sequential and discrete movement of ions through well‐defined crystallographic vacancies and interstitial sites.^[^ ^60^ ^]^ For example, argyrodite‐type Li_6_PS_5_X (X = Cl, Br, I) is known to show anion site disorder as promoting factor of lithium‐ion conductivity. In the cubic polymorph of Li_6_PS_5_X, anions form a face‐centered cubic (fcc) framework and S^2−^ and PS_4_^3−^ units occupy the octahedral and tetrahedral voids. The ionic conductivity increases with enhanced site disorder (highest in Li_6_PS_5_Cl: 60%). Correlated ion transport with halogen disorder was confirmed by combining solid‐state NMR and impedance measurements for Li_6_PS_5_Cl/Br SE by Hanghofer et al.^[^ ^61^ ^]^ The changes in anion distribution result in a modification of anionic charge, which influences lithium distribution. Additionally, the rotation of anionic species (PS_4_)^3−^ also promotes ion diffusion.^[^ ^61^ ^]^

SEB advantages include the absence of concentration polarization due to close to unity transference number of the SE, absence of solvated species and prevention of chemical interaction between the electrodes and the promise to produce safer cells with higher energy density as compared to LEBs. Noteworthy challenges in SEBs are crack formation and loss of contact at electrode‐SE interfaces due to volume expansion or interfacial reactions as well as dendritic growth causing shorting of the cell. For example, Tan et al. reported µSi as anode used in a µSi | Li_6_PS_5_Cl | NCM811 cell to overcome interface instability and current density limitations.^[^ ^62^ ^]^ Another important strategy to improve performance at higher current density is to fabricate SEBs with thicker cathode composite,^[^ ^63^ ^]^ high areal loading of cathode (e.g., 25.2 mg cm^−2^ for LiIn | Li_7_La_3_Zr_2_O_12_ | LiCoO_2_‐Li_7_La_3_Zr_2_O_12_),^[^ ^64^ ^]^ relative density as high as possible and thinner electrolyte for higher energy density. A new possibility was demonstrated of manufacturing sheet‐type multi‐stacked cells without applying pressure for enhancing energy density, especially with Ag/C composite, achieving 942 Wh l^−1^ with 10 parallel stacks of bi‐cells.^[^ ^65^ ^]^

For next‐generation SEBs, significant research is needed for anode development with nanostructured silicon anodes (e.g., columnar 3D silicon)^[^ ^66^ ^]^ or silver‐carbon anode composites as well as the development of conversion‐type and high‐voltage cathodes. New methods for manufacturing sheet‐type batteries with minimal binder content (<1 wt.%),^[^ ^67^ ^]^ new binder chemistries, and catholyte microstructures are important research topics to explore in more detail.^[^ ^68^ ^]^ In addition, scale‐up of cell fabrication and dry processing of materials and lithium metal and the requirement of applying pressure during cycling to maintain contact are key issues that remain to be solved on the way to commercialization of SEBs.

### 2.5 Hybrid Electrolyte Battery (HEB)

Hybridization of electrolytes opens the pathway to a manifold of subtypes of cells.^[^ ^69^ ^]^ In order to avoid complications with a plethora of combinations with individual names, we propose to simply classify all cells as HEBs that contain at least two electrolytes of different ion conduction mechanism. Therefore, there are four possible combinations: LE‐PE, LE‐SE, PE‐SE, LE‐PE‐SE, of which the PE‐SE combination is the most prominent case. These are mostly present in the HEB either in the form of a particle‐in‐matrix assembly or as separate electrolyte layers in a multilayer assembly (Figure 3d). Effective ion conduction through a HEB therefore requires effective transfer of lithium ions from one electrolyte type to another, which means the transfer from one ion conduction mechanism to another.[^21^] However, across the interface, ion transport depends on the interface structure and composition. Neither the SE‐LE nor the SE‐PE interfaces are without their challenges: both have been found to produce degradation products that hamper ion transfer.^[^ ^70^, ^71^ ^]^

#### 2.5.1 Particle‐In‐Matrix Assembly

Interspersed particles or fibers of SE in a matrix of another electrolyte type are characteristic of particle‐in‐matrix HEs, a prominent example being SE particles in a PE matrix, for example, the nanofiber Li_6.75_La_3_Zr_1.75_Ta_0.25_O_12_‐PEO‐LiTFSI HE with an overall ionic conductivity of 0.21 mS cm^−1^ at 25 °C.^[^ ^72^, ^73^ ^]^ Combining the mechanical properties of polymers with the high ionic conductivity of inorganic SEs to form a flexible sheet‐type mechanical consistency is desired with particle‐in‐matrix HEs.^[^ ^22^ ^]^

Particle‐in‐matrix HEs can be grouped into two cases: SE‐rich (>50 vol% SE content) and PE‐rich (<50 vol% SE content) HEs.^[^ ^15^ ^]^ For SE‐rich HEs, ion transport is guided by the SE phase, which typically has higher ionic conductivity than the PE phase. In PE‐rich particle‐in‐matrix HEs, unfortunately, the lithium transfer between PE and SE often has high resistance.^[^ ^74^ ^]^ Nonetheless, the presence of the inorganic phase reduces the crystallinity of polymers and increases the amorphous phase content and mobility of ions through the PE in this way.^[^ ^75^ ^]^ The advanced nanowire morphology of ceramic is incorporated into the polymer matrix to increase the ionic conductivity of the hybrid as compared to randomly distributed ceramic particles in a polymer.^[^ ^76^ ^]^ Alignment of the nanowire normal to the electrode results in enhancing conductivity at the ceramic/polymer interface.^[^ ^76^ ^]^ This explains the conduction at the macroscopic level.

At the microscopic level, ion transport between SE and PE phases in PEO‐LiTFSI‐Li_7_La_3_Zr_2_O_12_ hybrid electrolyte is predicted by Zagorski et al.^[^ ^22^ ^]^ The ion transport in such systems is guided mainly by polymer chain segmental mobility. The garnet filler does not contribute much to ion transport, as a high interfacial transfer resistance exists between the two phases. The interfacial stability of the SE‐PE interface influences the transference number. For example, in Li_3/8_Sr_7/16_Ta_3/4_Zr_1/4_O_3_‐PEO‐LiTFSI HEs, the interface traps anions to enhance lithium‐ion mobility, conductivity, and transference number.^[^ ^77^ ^]^ Another recent example is a hybrid integrating nanofibers of Li_1.3_Al_0.3_Ti_1.7_(PO_4_)_3_ (LATP) in single‐ion conducting PE lithium(4‐styrenesulfonyl)‐(trifluoromethanesulfonyl)imide (LiSTFSI) poly(bis(2‐(2‐methoxy ethoxy)ethoxy)‐phosphazene) (MEEP) for Li | LATP‐LiSTFSI‐MEEP | LiFePO_4_ reported by Yu et al.^[^ ^27^ ^]^

The incorporation of fillers into polymers enhances the mechanical strength compared with the pure polymer, which may aid in suppression of dendrite propagation and SEI composition at the anode favoring a stable SEI. For example, Chen et al. reported PEO‐LLZTO HEs that exhibit better dendrite suppression ability compared to the PE without filler particles.^[^ ^78^ ^]^ On the cathode side, the electrochemical window may be enhanced by fillers with high voltage electrolyte.^[^ ^73^ ^]^ Cell assembly of particle‐in‐matrix HEBs is similar to that of PEBs. The causes for performance enhancement by using highly conducting fillers are still under investigation and a careful evaluation is required for each case investigating the exact influence of fillers on the overall ionic conductivity.

#### 2.5.2 Multilayer Assembly

Multilayer HEs are formed if a hybrid electrode contains an electrolyte with a different conduction mechanism than the separator layer or if the separator layer itself is made of multiple layers of different electrolyte types. Intermixing of electrolytes is avoided and planar interfaces are typically obtained between the different electrolyte layers. For example, LEs or PEs with SE layers have been demonstrated, each concept pursuing different goals. Ion transport in multilayer HEs is often dominated by the transfer process across the interface. In addition, the chemical stability between the two electrolyte layers has a strong influence overall.

The LE‐SE combination typically is applied to protect a lithium metal anode using an oxide‐based SE while the LE acts as catholyte to ensure ionic transfer at the cathode interface. Fu et al. pioneered HEB development by presenting a lithium‐sulfur multilayer HEB using a dense‐porous bilayer of Li_7_La_2.75_Ca_0.25_Zr_1.75_Nb_0.25_O_12_ SE and pores infiltrated with sulfur and LE, achieving a HEB with 248 Wh kg^−1^.^[^ ^79^ ^]^ A dense layer of SE physically blocks dissolved polysulfides and lithium dendrite growth, which prevents degradation and shorting of the cell.^[^ ^80^ ^]^ Lithium dendrite penetration is hindered by the porous‐dense SE layer that protects lithium from polysulfide passivation, while a small amount of LE is used to ensure contact between the cathode and SE.^[^ ^81^ ^]^ A similar technology is pursued by QuantumScape using a SE to protect lithium metal and an organic GE as catholyte.^[^ ^82^ ^]^ Fuchs et al. used an ionic liquid electrolyte at the interface of garnet‐type SE and lithium metal to increase the stripping capacity of the lithium metal electrode by mitigating pore formation at the interface.^[^ ^83^ ^]^ While developing the LE‐SE multilayer system, a few crucial points are to be studied further, such as dissolution of ionic components into the LE, formation of a solid liquid electrolyte interface (SLEI) along the SE and LE interface, charge redistribution and potential difference between SE | LE, or decomposition reaction at the SLEI.^[^ ^84^ ^]^

For the PE‐SE combination, Zhou et al. observed improved Coulomb efficiency, better cycling performance, enhanced electrochemical stability, lower lithium transference number, and reduced anode/electrolyte interface resistance with negligible dendrite growth in Li | c‐l‐PA | LATP | LiFePO_4_ cells (c‐l‐PA: crosslinked polyacrylate main chain and oligo ethylene oxide side chains) as compared to a corresponding Li | c‐l‐PA | LiFePO_4_ cell.^[^ ^85^ ^]^ The authors proposed that the anion‐blocking nature of LATP improved the overall transference number. Similarly, Ates et al. presented cells in which a lithium metal anode is protected from reaction with Li_3_PS_4_ SE by a PEO‐LiTFSI PE interlayer.^[^ ^86^ ^]^ Ionic transport across the interface was studied in Li | PEO‐LiTFSI | Li_6_PS_5_Cl | PEO‐LiTFSI | Li cells using reference electrodes, concluding lower interface resistance for charge transfer compared to oxide interface.^[^ ^71^ ^]^ The polymer interlayers reduce interfacial degradation between lithium and argyrodite in this multilayer HE design. The polymer interlayer on LATP SE is explored to report a Li | PE‐SE‐PE | Li_3_V_2_(PO_4_)_3_/CNT cell, which delivers high specific energy based on the weight of the electrodes (460 Wh Kg^−1^).^[^ ^87^ ^]^ Here, the PE enables a lower external pressure for operation and wider electrochemical window and high energy density with lithium metal. The drawback of this cell is their poor cyclability as compared to the LE due to poor conductivity and lower contact area between electrode and electrolyte. In terms of scalability of manufacture, wet coating, screen printing and tape casting methods are feasible to scale up HEBs with tape casted SE and PEO‐LiTFSI as reported by Ates et al. (Li‐In | PEO‐LiTFSI | β‐Li_3_PS_4_ | NMC622‐Li_3_PS_4_‐VGCF).^[^ ^86^ ^]^

### 2.6 Discussion of Ambiguous Cases

With the advent of hybridized cell concepts, it is sometimes not immediately evident, which classification is appropriate. Therefore, we summarize several ambiguous cases in this section. Nonetheless, we encourage all authors of such investigations to identify the appropriate classification for their respective cells. For example, unconventional ion transport is observed in superconcentrated electrolytes, such as glyme‐, or sulfolane‐based electrolytes,^[^ ^88^ ^]^ where ions move between aggregated ion pairs.^[^ ^73^ ^]^ Similarly, when rigid polymers (polyether, polycarbonate, PEO, polyacrylonitrile) are doped with large amount of salt (0.1–0.2 unit of polyether per salt unit), close packing of rigid polymer chains provides large free volume for ion motion, resulting in ion transport decoupled from polymer segmental motion reaching a conductivity of 10^−4^ S cm^−1^ in this concentrated regime or for polyionic liquids and liquid crystalline conductors.^[^ ^89^ ^]^

Another complex case are liquid crystal and plastic crystal electrolytes (e.g., Li_2_SO_4_, succinonitrile, ionic liquid),^[^ ^90^ ^]^ where ion transport is guided by the rotational dynamics of the molecules within their plastic crystal phase (below the melting point).^[^ ^91^ ^]^ Here, we recommend that distinction between a SE and a LE is made on a case‐by‐case basis, dependent on the predominant ion‐conduction mechanism at the operation temperature of the battery cell. Sen et al.^[^ ^92^ ^]^ presented hyperbranched dendrimer electrolytes achieving near to unity transference number in the liquid state. The immobilization of TFSI^−^ by the branched peripheral functional group decreases anion diffusion and increases the transference number for lithium ions. Even though the electrolyte contains solubilized polymers, the main mode of ion conduction is via the LE and therefore this material combination is part of the LE typology.

Similarly, soggy sand electrolytes^[^ ^93^ ^]^ are a dispersion of solid particles (SiO_2_, Al_2_O_3_, etc.) in a LE matrix (e.g., LiClO_4_ in MeOH).^[^ ^94^ ^]^ The presence of non‐conducting filler particles increases the ionic conductivity in the liquid phase by altering cation and anion diffusion at the interface between inorganic filler particles and the liquid matrix. As the mechanical properties change from liquid to gel with increasing particle content, soggy sand electrolytes may be classified as either a LE or GE. If the embedded particles themselves are SEs, then a HE is formed. The same is true for particle‐in‐matrix HEs of SE particles in a PE matrix, which together form a HE due to the presence of two different ion‐conduction mechanisms. In contrast, the embedding of non‐conducting particles in a PE matrix simply classifies this combination as a PPE because non‐conducting fillers serve to increase the ionic conductivity of the PE.

One interesting exception in the conduction mechanism of polymer‐based ion‐conductors is reported by Gadjourova et al. in the PEO‐LiAsF_6_ (6:1 complex).^[^ ^95^ ^]^ The PEO chain forms a tunnel in conjunction with lithium coordination and lithium ions can move through the crystalline phase, which makes these crystalline polymer‐based ion‐conductors part of the SE group.

Finally, based on our proposed method of classification, the presence of a non‐conducting polymer in a 3D bicontinuous scaffold of a SE results, simply, as a SE.^[^ ^7^ ^]^ In addition, the combination of two different SEs in a multilayer assembly simply makes a SEB, as ion conduction via vacancies and interstitial sites is fundamental to both SEs. Such a multilayer assembly of SEs may therefore be distinguished as a multilayer SEB. This is applicable independent of whether the two SEs are both sulfur‐based,^[^ ^96^ ^]^ or whether one SE is sulfur‐based and the other is oxide‐based.^[^ ^97^ ^]^

### 2.7 Unified Full Cell Typology with Anode and Cathode Active Materials

**Figure 4:**
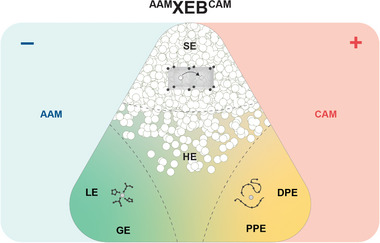
Overview of the full cell typology ^AAM^XEB^CAM^ based on the conduction mechanism of the electrolyte.

The final point regarding battery notation addresses the question, of how to represent the full battery chemistry including electrodes and electrolyte in a simple and unified manner. Typically, electrochemical cells are represented as negative electrode | electrolyte | positive electrode. For a more pragmatic representation of battery cells, a shorter notation is desired. Here, we propose ^AAM^XEB^CAM^ where AAM is the anode active material (negative electrode), XEB stands for LEB, GEB, PPEB, DPEB, SEB or HEB and CAM is the cathode active material (positive electrode), see **Figure** **4**. For example, in the case of a lithium‐ion battery with graphite (C), liquid electrolyte (LE) and LiCoO_2_ (LCO), the notation is simply ^C^LEB^LCO^.

Further examples are listed in **Table** **1** along with selected cell performance measures. Typical AAMs for lithium batteries are lithium metal (Li), graphite (C), silicon (Si), indium‐lithium alloy (InLi), or Li_4_Ti_5_O_12_ (LTO). The special case of so‐called anode‐free, zero‐excess lithium, or lithium reservoir free cells may be represented by a 0, for example ^0^SEB^NMC^. Typical CAMs for lithium batteries are LiCoO_2_ (LCO), LiNi_x_Mn_y_Co_z_O_2_ (NMC), LiFePO_4_ (LFP), sulfur (S) or lithium sulfide (Li2S) (dependent on which material is used to build the cell), oxygen (O2) or air (air).

**Table 1:** Composition and performance measures for different types of battery cells (data includes cell casing if marked with an asterisk).

Notation	AAM	Separator	CAM	Cycling temp./°C	Current density/mA cm^−2^	Area capacity/mAh cm^−2^	Specific energy/Wh kg^−1^	Energy density/Wh l^−1^	Ref.
^Li^LEB^NMC^	Li	LE	NMC	25	1	10	711^a)^	1654^a)^	[98]
^C^LEB^NCA^	C	LE	NCA	25	ND	ND	243^a)^	676^a)^	[99]
^Li^GEB^NMC^	Li	GE	NMC	25	0.1	10.3	410	ND	[40]
^Li^DPEB^LFP^	Li	PEO‐LiTFSI	LFP	70	0.81	2.3	282^b)^	ND	[45]
^Li^SEB^CuS^	Li	Li_3_PS_4_	CuS	25	0.43	4.9	58	ND	[100]
^0^SEB^NMC^	0	Li_6_PS_5_Cl	NMC	25	0.68	6.8	ND	942^a)^	[65]
multilayer ^Li^HEB^S^	Li	Li_7_La_3_Zr_2_O_12_ + LE	S	25	0.2	12.6	248	ND	[79]
^Na^LEB^NCFMO^	Na	LE	NCFMO^c)^	25	≈1.5	≈3	205	ND	[101]
^Si^SEB^NMC^	Si	Li_6_PS_5_Cl	NMC	ND	0.4	4.0	266	ND	[102]

^a)^ includes cell casing;

^b)^ value estimated using Supplementary data 1 of Randau et al.^[^ ^2^ ^]^;

^c)^ NCFMO: Na[Cu_1/9_Ni_2/9_Fe_1/3_Mn_1/3_]O_2_.

In order to represent the large variety of active material concepts in short form, the corresponding superscript may also point out a specific subtype of AAM or CAM. For example, the use of specially designed silicon nanoparticles (nanoSi), or silicon thin films (2DSi), a certain composition like LiNi_0.8_Co_0.1_Mn_0.1_O_2_ (NMC811), or morphology like single crystal NMC (scNMC) may be indicated as such. The same is applicable to blends of active materials. For example, the use of blended silicon and graphite (Si,C) electrodes or blended LCO and NMC (LCO,NMC) electrodes may be specified using a comma as in this example: ^Si,C^LEB^LCO,NMC^. If desired, specific sub‐cases of each battery type may be specified effectively with an additional prefix such as aqueous LEB, sodium metal LEB, argyrodyte SEB, particle‐in‐matrix HEB, multilayer SEB, blended anode LEB, nanostructured HEB etc.

Even though the proposed notation originates out of considerations from lithium battery research, in principle, any type of battery may be represented thereby, as exemplified by the following examples: a typical lead‐acid battery may be noted as ^Pb^LEB^PbO2^, or a zinc‐air battery may be noted as ^Zn^LEB^air^. High‐temperature sodium‐sulfur batteries that use a SE (for example, sodium beta alumina) and liquid active materials may be termed ^Na(l)^SEB^S(l)^, which allows to distinguish them from a ^Na^SEB^S^ that runs at temperatures below the melting point of the active materials. Finally, a note on electrochemical cells that are not considered here due to their different working principle and application perspective: redox‐flow batteries and fuel cells. Our proposed terminology focuses on batteries relying on immobile electrodes without additional external storage tanks and pumps.

### 2.8 Cell Performance Evaluation

The cell performance measures listed in Table 1 for several examples of different cell types demonstrate that all have been developed to an advanced level, but that cell data isn't always reported completely, which impedes a thorough and systematic comparison. Even though the ionic conductivity achieved with LEs and SEs is similar, the comparative ease of processing of cells with LEs means that LEBs are a moving target for the other cell types, which are also progressing rapidly. While the other cell types mature to the level of LEBs, it is expected that development, testing and data evaluation protocols will align increasingly, enabling a more stringent and systematic comparison of battery cell performance across the board. For detailed information on each cell type, we refer to the respective review articles.

In general, the most important features of battery performance evaluation may be described as follows. Independent of the cell type, it is necessary to combine scientific advance on a fundamental level with advances in cell engineering and processing technology to enable the fabrication of a battery cell with improved overall performance over today's established industrial processes and commercial cells. The focus of fundamental research lies on materials innovation, interface modification, metal anode development, new cell chemistries, battery safety etc. This is particularly relevant for the synthesis of better‐performing CAMs, electrolytes and cathode composites with higher energy storage capability (potential, capacity), faster rate capability (conductivity, internal resistance) and longer cycling stability (energy retention, Coulomb efficiency). In addition, the focus of engineering and processing lies on maximizing the CAM content and minimizing the content of all other cell components by decreasing separator thickness, current collector thickness, electrolyte content, cell casing etc.

Arguably, this means that the CAM is the central cell component as it is the only one that is to be maximized in order to increase the energy density of the cell. In contrast, the corresponding metal is usually the ideal AAM and may in principle be formed in situ during charging of the cell using the metal ions contained in the CAM when building the cell. Also, the content of all other cell components is to be minimized to increase the energy density, as they do not contribute to the storage of energy, but each serves specific purposes required for the functioning and power capability of the cell.

Finding the compromise in balancing the energy storage capability and power capability lies at the heart of battery research, design and development. This dichotomy makes a simple and yet comprehensive comparison of the performance of different cells quite challenging, as both of these performance measures are mutually dependent and may each be increased at the cost of the other. The Ragone diagram provides a suitable tool for evaluating both energy storage and power output in one graph and is applicable to all cell types. Unfortunately, not all parameters required for this evaluation are comprehensively reported in the literature. In order to better address this issue, Randau et al. proposed a minimum set of cell and cycling parameters from which all other performance measures can be calculated.^[^ ^2^ ^]^ Correspondingly, we anticipate that more comprehensive and uniform reporting of the indicated minimum set of parameters and performance measures will expedite the exchange of results across all cell types.

## 3 Conclusion

In summary, we propose a uniform cell typology that is centered around the ion conduction mechanism of the electrolyte. For selecting the appropriate electrolyte to achieve commercial success of a battery cell, it is important to have a clear understanding of the different types of electrolytes available. Similarly, for evaluating the academic progress of cells over the state‐of‐the‐art within their respective categories, it is fundamental that cells are assigned clearly to a certain cell type, as their respective technology readiness levels are different. Therefore, we review each battery type briefly and propose a practical and systematic classification method that can be applied, in principle, to all types of battery cells, as it is based on the predominant ion conduction mechanism of the electrolyte. This uniform and effective method of categorizing batteries also enables a short notation with which to specify the internal cell chemistry briefly: ^AAM^XEB^CAM^. We anticipate that its adoption will aid with clarity, focus and building bridges between different strands of battery research.

## Conflict of Interest

The authors declare no conflict of interest.
